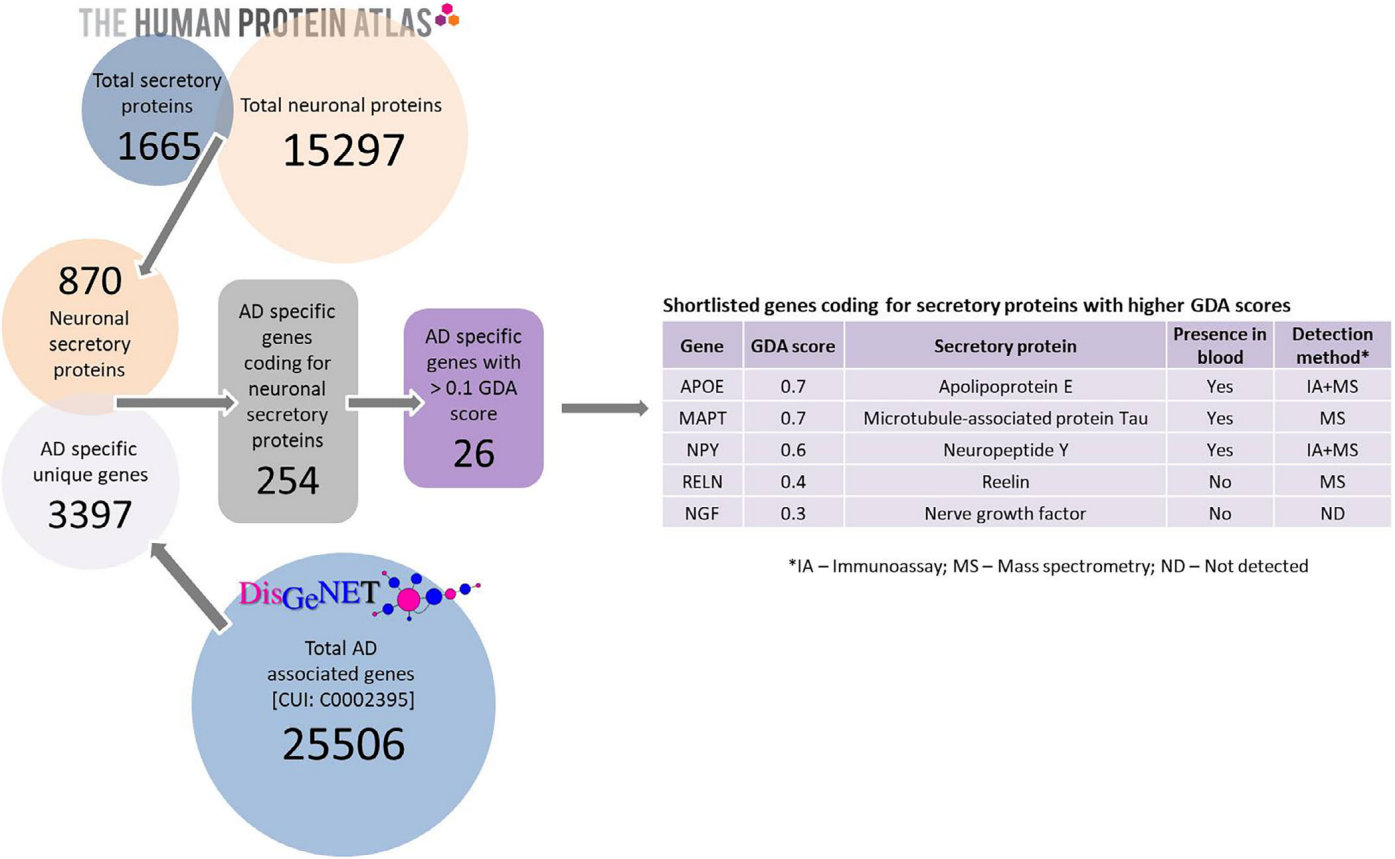# A Study on the Expression Profile of Genes Coding for Secretory Proteins Using in vitro Model of Alzheimer’s Disease

**DOI:** 10.1002/alz.092209

**Published:** 2025-01-09

**Authors:** Ravinder Singh, Aditya Sunkaria

**Affiliations:** ^1^ Guru Nanak Dev University, Amritsar, Punjab India

## Abstract

**Background:**

Age‐related neurological illness like Alzheimer's disease (AD) is steadily becoming more prevalent among the aged population in India and around the world. Cognitive deficits are caused by a progressive loss of normal brain functions. Increased production of amyloid (Aβ) and the development of neurofibrillary tangles (NFTs) are the two most significant pathogenic events that take place during AD progression. Until today, many attempts have been made towards discovering novel targets which are more precise and could identify the progression of the disease at very early stage.

**Method:**

In the present study the Human Protein Atlas (HPA) database was used to filter out total human secretome. Using single cell type proteome of the HPA database, neuronal proteins were compared with total human secretome to discover overlapping proteins and their respective genes. Further, Alzheimer's disease associated genes were extracted using DisGeNet database and ranked according to their gene‐disease association (gda) score. Further, different proteoforms of Aβ were used to treat neuronal cells in vitro to mimic early stages of AD like pathology. The expression of secretory protein genes from in vitro model system has been analysed.

**Result:**

Out of 1678 genes, 24 neuronal genes were identified that were coding for secretory proteins. These genes were further categorized according to their proteins function. Specific primers for the top gda score genes were designed and expression analyses were conducted. Analyses revealed modulation in the expression of APOE, MAPT, NPY, NGF, and RELN genes.

**Conclusion:**

The aim of this study was to discover early‐stage biomarkers for Alzheimer's disease. In the present study we have tried to identify modulation in the genes coding for secretory proteins which might be reflected at protein levels also. Currently our lab is working on to fully understand the modulation of AD secretome which would enable us to identify AD specific early stage biomarkers.